# Enhanced Low-Frequency Sound Absorption of a Porous Layer Mosaicked with Perforated Resonator

**DOI:** 10.3390/polym14020223

**Published:** 2022-01-06

**Authors:** Xin Li, Bilong Liu, Qianqian Wu

**Affiliations:** 1 School of Mechanical & Automobile Engineering, Qingdao University of Technology, No. 777 Jialingjiang Road, Qingdao 266520, China; jz03-4lx@163.com (X.L.); wuqq2012@126.com (Q.W.); 2 Key Lab of Industrial Fluid Energy Conservation and Pollution Control, Qingdao University of Technology, Ministry of Education, No. 777 Jialingjiang Road, Qingdao 266520, China

**Keywords:** perforated resonator, porous materials, combined effect, sound absorption enhancement

## Abstract

A composite structure composed of a porous-material layer mosaicked with a perforated resonator is proposed to improve the low-frequency sound absorption of the porous layer. This structure is investigated in the form of a porous-material matrix (PM) and a perforated resonator (PR), and the PR is a thin perforated plate filled with porous material in its back cavity. Theoretical and numerical models are established to predict the acoustic impedance and sound absorption coefficient of the proposed structure, and two samples made of polyurethane and melamine, respectively, are tested in an impedance tube. The predicted results are consistent with that of the measured. Compared with a single porous layer with the same thickness, the results show that the designed structure provides an additional sound absorption peak at low frequencies. The proposed structure is compact and has an effective absorption bandwidth of more than two octaves especially below the frequency corresponding to 1/4 wavelength. A comparison is also made between the sound absorption coefficients of the proposed structure and a classical micro-perforated plate (MPP), and the results reveal equivalent acoustic performance, suggesting that it can be used as an alternative to the MPP for low–mid frequency sound absorption. Moreover, the influences of the main parameters on the sound absorption coefficient of PPCS are also analyzed, such as the hole diameter, area ratio, flow resistance, and porous-material thickness in the PR. The mechanism of sound absorption is discussed through the surface acoustic impedance and the distributions of particle velocity and sound pressure at several specific frequencies. This work provides a new idea for the applications of the thin porous layer in low- and medium-frequency sound absorption.

## 1. Introduction

Porous materials are usually composed of two phases, i.e., solid framework interwoven with pore network, and in the vicinity of the solid–air interface, the sound energy is consumed through viscous dissipation and heat conduction [1,2,3]. According to the famous Stokes–Kirchhoff formula, the energy dissipation rate is proportional to the quadratic frequency, so porous materials can effectively absorb the acoustic waves at medium and high frequencies in practical applications. To achieve satisfactory sound absorption, the minimum thickness of a porous material should generally be no less than 1/4 wavelength, and this requirement constrains their wide applications in low-frequency sound absorption, especially in limited spaces.

Many studies have been concerned about enhancing the low-frequency sound absorption of porous materials. In earlier years, perforated plates in the form of complete perforated facings [4,5,6,7] covering the front of porous materials can bring a relatively larger absorption bandwidth at low frequencies, especially those perforated plates with small perforation rates, although inevitably at the cost of lower high-frequency absorption than the pure porous materials. With the development of micro-perforated panels (MPP) theory [8,9], stretched ceilings based on micro-perforated panels [10,11] have been successfully used in room acoustics, and the sound absorption performance of MPP stretched ceilings filled with porous material is better than that of MPP with only air in the cavity. Later, Sakagami et al. [12] investigated the sound absorption characteristics of a single MPP absorber with a back cavity completely filled with porous material and established a theoretical acoustic impedance model using the electro-acoustical equivalent method. In addition, for the serial structure consisting of MPP, airspace, and porous material, Li et al. [13] gave a theoretical prediction model with the transfer matrix method, and comparison of different results showed that the sound absorption performance of “porous material–air layer–MPP” is better than that of “MPP–air–porous material”. The porous material placed in front of the MPP helps to absorb high-frequency sound waves, and a large thickness is required for absorbing low-frequency sound waves. Parallel devices with porous materials [14,15,16,17] have also been studied to achieve effective sound absorption at low–mid frequencies in a limited space. A parallel arrangement of three perforated plates with extended tubes (PPETs) and porous materials was used to improve the sound absorption in the frequency range of 100–1600 Hz with a limited total thickness of 100 mm [14]. A structure composed of porous materials and common perforated plate resonators with low perforation rate was designed by Li et al. [16] based on the principle of acoustic impedance matching, and an L-shaped cavity was used to provide more compliance. This structure with a total thickness of 80 mm has an average absorption coefficient of 0.9 in the frequency range of 200–1600 Hz.

Recently, a new concept has been proposed for sound absorptive materials with double porosities. Different from the conventional materials with a single porosity, double-porosity materials consisting of two interconnected networks of pores with different characteristic sizes are recognized as meso-perforated materials [18,19,20]. Boutin et al. [18] derived a macroscopic description of sound propagation through double-porosity materials using the homogenization method for periodic structures (HPS) in the scope of geophysics applications. Olny et al. [19] further applied this theory to realistic double-porosity materials with static skeleton and elucidated that the macroscopic descriptions of sound propagation depend on the contrast of static permeability between pores and micropores and also on the frequency. Sgard et al. [20] designed a double-porosity material by perforating straight holes or holes with cross-sectional variation in a porous-material layer, and the results showed that the use of perforations can enhance the sound absorption of the porous material in a selected frequency range. Based on the HPS theory, more double-porosity materials were proposed to improve the sound absorption of conventional porous materials based on the coupling effect of micropores and mesopores, such as slit perforated porous material [21], gradually perforated porous materials [22], porous material with labyrinthine channel [23], and two different micro-porous materials [24].

Another type of sound absorption enhancement has a composite structure with resonant inclusions embedded in the porous material matrix [25,26,27,28,29,30,31,32,33]. Groby et al. [28] studied the absorption properties of a metaporous material made of non-resonant three-dimensional rigid inclusions embedded in a rigidly backed rigid-frame porous material. The shapes of the rigid simple inclusions are cube, cylinder, sphere, cone, and ring torus. The results showed that the cube can achieve better sound absorption enhancement at low frequencies due to its larger filling fraction. Later, Groby et al. [30] numerically and experimentally studied the sound absorption of a thin porous foam layer with periodically embedded Helmholtz resonators (HR), and the results showed that when the structure was well designed, two absorption peaks appeared at low frequencies originating from two Helmholtz resonators. Zhu et al. [31] designed an ultra-thin absorber with four acoustic metamaterial resonators inserted in a porous-material layer, and a large sound absorption coefficient (larger than 0.8) was obtained in the frequency range of 180–550 Hz. It was demonstrated that the enhancement of sound absorption at frequencies lower than a quarter wavelength results from the Helmholtz resonance in the viscous regime and the trapped modes in the inertial regime. In addition, with the maturity of rapid additive manufacturing technology, the optimized graded porous materials [34,35], folded metaporous material [36], and anisotropic metaporous surfaces [37] have been fabricated using 3D printing for broadband sound absorption.

Although a great deal of research has been conducted in the past on the sound absorptive properties of porous materials, it is still necessary to improve the low-frequency sound absorption performance of porous materials, especially for compact acoustic structures in the control of noise in the low-to-mid frequency range. In this paper, a new composite structure is proposed by mosaicking a perforated resonator in a layer of porous material, which is abbreviated as PPCS. The benefit of the PPCS is that it produces an additional absorption peak at lower frequencies in comparison with that of a single layer of the same porous material with the same thickness. Firstly, a theoretical model and a numerical model are constructed to predict the acoustic properties of PPCS, and the accuracies of both models are checked by comparing the calculated results with the measured results obtained in an impedance tube. Then, the underlying mechanism of the sound absorption of PPCS is discussed through the surface acoustic impedances and the distributions of particle velocity and sound pressure at specific frequencies. A comparison made between the sound absorption coefficients of PPCS and MPP is provided. Finally, the influences of the following parameters are discussed on the sound absorption performance of PPCS: the hole diameter, the area occupancy ratio, the thickness of porous material in the perforated resonator (PR), and flow resistance in the porous material (PM).

## 2. Models and Methods

### 2.1. Theoretical Model of PPCS

**Figure 1:**
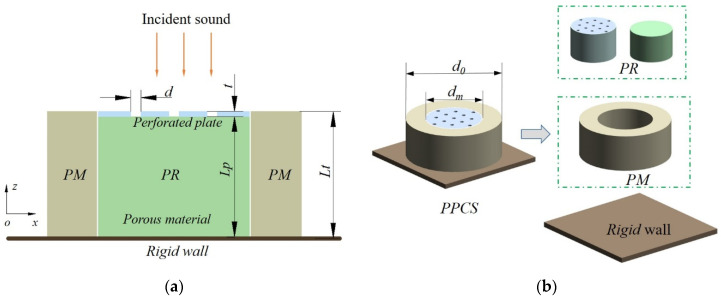
Schematic diagrams of the PPCS. (**a**) Two-dimensional cross-sectional view; (**b**) three-dimensional assembly view.

Figure 1 shows the cross-section of a PPCS absorber in the “xoz” coordinates under the incidence of sound waves along the negative *z*-axis. The absorber consists of a layer of porous material (PM) and a perforated resonator (PR) arranged in parallel on a rigid wall. The PR is a resonant structure composed of a rigid perforated plate and back cavity filled with porous material (the same material is analyzed and used in this paper). The two white solid lines in Figure 1a mark the rigid interface between the PM and PR components. The symbol *L_t_* is the total thickness of the PPCS, which is also the thickness of the porous-material layer; *L_p_* is the thickness of porous material in the PR and also is the distance between the perforated plate and rigid wall; *t* and *d* are the thickness of the perforated plate and the hole diameter, respectively. Given that a cylindrical impedance tube is used for measurement, the PM component here is designed as a cylinder with a hollow column at the center, and the PR component is composed of a circular perforated plate and a column cavity filled with a porous-material layer, as illustrated in Figure 1b. The outer and inner diameters of the porous-material cylinder are *d*_0_ and *d_m_*, respectively, and the outer diameter of the cylinder is the same as the inner diameter of the PM.

According to the electro-acoustical theory, the PPCS is analogous to a parallel circuit composed of two branches corresponding to the PM and PR. Therefore, the relationship between the surface acoustic impedance of PPCS and the two components (PM and PR) satisfies

(1) $$\frac{A_{0}}{Z_{PPCS}}=\frac{A_{PM}}{Z_{PM}}+\frac{A_{PR}}{Z_{PR}}$$

where $Z_{PPCS}$, $Z_{PM}$, and $Z_{PR}$ are the acoustic impedances of the PPCS, PM and PR, respectively; $A_{0}=\pi d_{0}{}^{2}/4$, $A_{PM}=\pi (d_{0}{}^{2}-d_{m}{}^{2})/4$, and $A_{PR}=\pi {\left(d_{m}-2t_{p}\right)}^{2}/4$ are the transverse cross-sectional areas of the PPCS, PM and PR, respectively; $t_{p}$ is the wall thickness of the PR cylinder.

The reflection coefficient at the surface of the PPCS is written as

(2) $$R=\frac{Z_{PPCS}-\rho_{0}c_{0}}{Z_{PPCS}+\rho_{0}c_{0}}$$

where $\rho_{0}$ and $c_{0}$ are the air density and the speed of sound propagation in the air, respectively. Then, the absorption coefficient of the PPCS at normal incidence is defined as

(3) $$\alpha =1-{\left|R\right|}^{2}$$

The five-parameter JCA model is known as a semi-empirical model that effectively describes the characteristics of acoustic propagation in porous materials with motionless skeletons over a wide range of frequencies [38,39]. The porous materials used in this paper are common melamine and polyurethane, which are generally treated as motionless-skeleton porous materials. Johnson et al. [40] proposed a semi-phenomenological model to describe the complex density of an acoustical porous material with a motionless skeleton having arbitrary pore shapes, and the equivalent dynamic density $\rho_{porous}$ is expressed as

(4) $$\rho_{porous}=\frac{\rho_{0}\alpha_{\infty }}{\phi }\left(1+\frac{\phi \sigma }{j\omega \rho_{0}\alpha_{\infty }}{\left(1+j\frac{4\omega \rho_{0}\eta \alpha_{\infty }{}^{2}}{{\left(\sigma \phi \Lambda \right)}^{2}}\right)}^{1/2}\right)$$

Champoux and Allard [41] introduced an expression for the dynamic bulk modulus for the same kind of porous material, and the equivalent bulk modulus $K_{porous}$ is expressed as

(5) $$K_{porous}=\frac{\gamma P_{0}}{\phi }{\left(\gamma -\left(\gamma -1\right){\left(1+\frac{8\eta }{j\omega \mathrm{Pr}\Lambda^{\prime 2}\rho_{0}}{\left(1+j\frac{\omega N_{Pr}\rho_{0}\Lambda^{\prime 2}}{16\eta }\right)}^{1/2}\right)}^{-1}\right)}^{-1}$$

In Equations (4) and (5), the equivalent dynamic density and bulk modulus are defined through five non-acoustic parameters of the porous material: the static flow resistance σ, porosity ϕ, the tortuosity $\alpha_{\infty }$, the viscous characteristic length Λ, and the thermal characteristic length $\Lambda^{\prime }$; ω is the angular frequency; η, γ and Pr are the dynamic viscosity, specific heat ratio and Prandtl number of the saturated air, respectively; $P_{0}$ is the ambient atmospheric pressure.

Then, the characteristic acoustic impedance $z_{p}$ and the complex wavenumber $k_{p}$ of a porous material with motionless skeletons are written as

(6) $$z_{p}=\sqrt{\rho_{porous}K_{porous}}$$

(7) $$k_{p}=\omega \sqrt{{\rho_{porous}/K}_{porous}}$$

The transfer matrix of the porous-material layer in this paper is expressed as

(8) $$T_{porous}=\left[\begin{array}{cc} \cos(k_{p}L_{p}) & jz_{p}\sin(k_{p}L_{p})\\ j\sin(k_{p}L_{p})/z_{p} & \cos(k_{p}L_{p}) \end{array}\right]$$

where *L_t_* is the thickness of this layer. It is backed by a rigid wall, so the surface impedance is expressed as

(9) $$Z_{PM}=-jz_{p}\cot(k_{p}L_{t})$$

Applying the fluid dynamic equations to the propagation of acoustic waves in a circular tube according to the Rayleigh theory [8], the acoustic impedance in the tube is expressed as

(10) $$z_{\mathrm{tube}}=j\omega \rho_{0}t{\left[1-\frac{2}{k\sqrt{-j}}\frac{J_{1}\left(k\sqrt{-j}\right)}{J_{0}\left(k\sqrt{-j}\right)}\right]}^{-1}$$

where $k=d/2\sqrt{\omega \rho_{0}/\eta }$ is a dimensionless number and $\sqrt{\eta /\omega \rho_{0}}$ is the thickness of viscous boundary layer; $J_{0}$ and $J_{1}$ are the Bessel functions of the order 0 and 1, respectively.

The perforated plate is considered to be a series of short tubes arranged in parallel. For a perforated plate with a perforation ratio *p*, the acoustic impedance is defined as the acoustic impedance of individual short tubes divided by the perforation ratio. Apply a simple approximation formula applicable to all values of *k*, and the acoustic impedance of the perforated plate can be written as [8]

(11) $$z_{pp}=\frac{32\eta t}{pd^{2}}\sqrt{1+\frac{k^{2}}{32}}+j\omega \rho_{0}t\left[1+{\left(3^{2}+\frac{k^{2}}{2}\right)}^{-1/2}\right]$$

In addition, considering the dissipation of sound energy near the inlet and outlet of the orifice and the piston emission at both ends, the correction of the acoustic impedance is expressed as

(12) $$\Delta z=\Delta r+j\omega \Delta m=\sqrt{0.5\omega \rho_{0}\eta }+j0.85\omega d$$

Therefore, the total acoustic impedance of the perforated plate includes the acoustic impedance in the tube and the end correction, which is expressed as

(13) $$Z_{pp}=\frac{32\eta t}{pd^{2}}\left(\sqrt{1+\frac{k^{2}}{32}}+\frac{\sqrt{2}}{32}k\frac{d}{t}\right)+\frac{j\omega \rho_{0}t}{p}\left[1+{\left(9+\frac{k^{2}}{2}\right)}^{-1/2}+0.85\frac{d}{t}\right]$$

Similarly, the transfer matrix of the perforated plate is expressed as

(14) $$T_{pp}=\left[\begin{array}{cc} 1 & Z_{pp}\\ 0 & 1 \end{array}\right]$$

Such a perforated plate connected in series with a porous-material layer constitutes the PR component. According to the theory of impedance transfer, the transfer matrix of the PR is expressed as

(15) $$T_{PR}=T_{pp}\cdot T_{porous}$$

Thus, the surface acoustic impedance of the PR backed by a rigid wall is derived as

(16) $$Z_{PR}=Z_{pp}-jz_{p}\cot(k_{p}L_{p})$$

### 2.2. Numerical Model of PPCS

**Figure 2:**
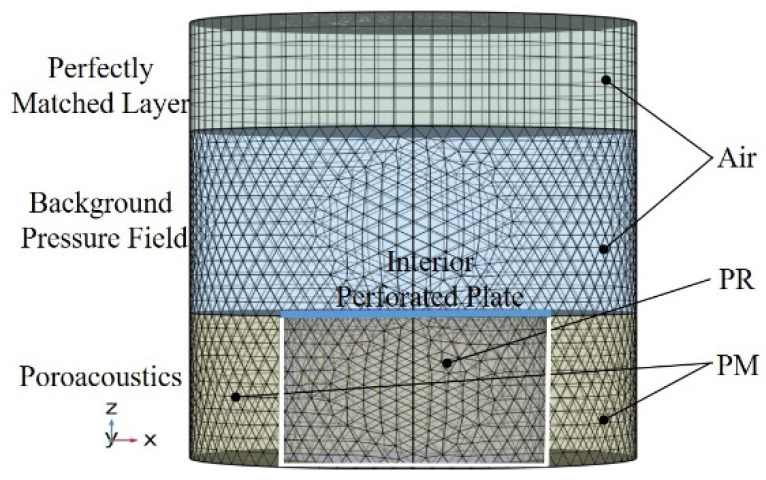
The finite element model of the PPCS.

As the main components of the designed structure are composed of porous materials and common perforated plate, a 3D numerical model is constructed to simulate the test sample in the pressure acoustic domain of the commercial finite element software Comsol Multiphysics. As shown in Figure 2, this finite element model consists of four parts from top to bottom: the perfectly matched layer, background pressure field, interior perforated plate, and poroacoustics. The perfectly matched layer is used to create an infinite air domain, which makes the upper boundary of the background sound field free of spurious reflections. The background pressure field represents the incident wave, which is a plane wave along the negative *z*-axis. The “Interior Perforated Plate” node in the software is used to define the transfer impedance of the perforated plate with round holes, and the “Thick Plate” model type is chosen to include the thermal effect. The poroacoustics part simulates based on the equivalent fluid theory the attenuation and dispersion of pressure waves as they propagate through porous materials, and the porous material in the PR and PM are described by the “Johnson–Champoux–Allard model” provided by the software. In addition, to obtain an accurate result, the maximum mesh size of the model is set to be smaller than one-sixth of the minimum wavelength. The Free Tetrahedral meshes are created in the background sound field and structural domain of the PR and PM, and the swept mode is applied in the perfect match layer.

Then, the surface acoustic impedance and sound-pressure reflection coefficient of the structure are expressed as

(17) $$Z=\frac{p}{u_{n}}$$

(18) $$R=\frac{p_{sca}}{p_{in}}$$

where p, $p_{sca}$, and $p_{in}$ denote the total, incident, and scattered sound pressure, respectively, and $u_{n}$ is the particle velocity on the surface of the structure. Hence, the sound absorption coefficient of the PPCS can be obtained according to Equation (3).

### 2.3. Experimental Validation

**Figure 3:**
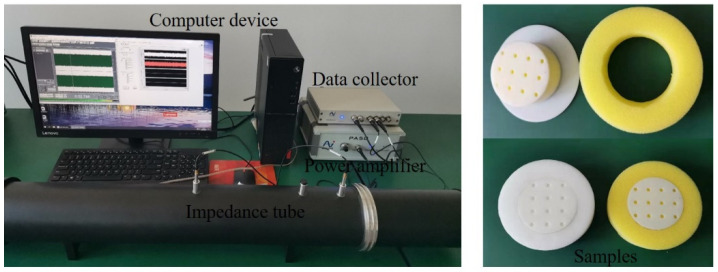
Experimental equipment and samples.

To further verify the feasibility of the proposed prediction methods, the normal sound absorption coefficients of two PPCS samples are measured using an SW422 impedance tube system with the transfer function method in ISO 10534-2 [42]. As shown in Figure 3, an impedance tube with a diameter of 100 mm is used for sound absorption measurement in the frequency range of 63–1600 Hz. For the convenience of sample mounting, the outer diameter of the prepared sample is approximately 99.5 mm. The PM component is formed by removing excess material with a circular cutter, and the PR is fabricated via 3D printing using epoxy resin materials. In addition, the sample and the inner wall of the tube can be sealed with PTFE film tape to ensure accurate installation. The acoustic impedance of the epoxy resin material is greater than that of the air, so the PR is regarded as a rigid structure. Here, common porous materials such as melamine foam and polyurethane are tested in the experiments, and the characteristic parameters of these porous materials are obtained with the inversion method based on impedance tube measurement [39]. The approximate structural parameters of the PPCS samples are shown in Table 1.

**Figure 4:**
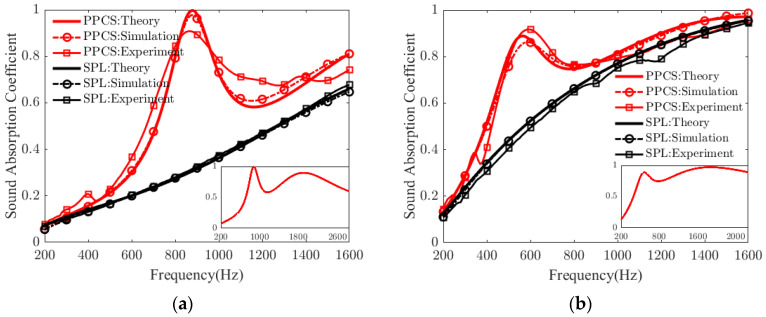
Sound absorption coefficients of PPCS under theory, simulation, and experiment. (**a**) Porous materials of melamine; (**b**) porous materials of polyurethane.

The measured sound absorption coefficients of the PPCSs with PMs made of melamine and polyurethane are shown in Figure 4a,b, respectively. For the PPCS and a single porous layer (SPL), the sound absorption coefficients calculated by the theoretical model are in good agreement with those simulated by the numerical model. These experimental results are consistent with the predicted results obtained in both ways, with some deviations mostly likely caused by the inaccurate mounting of the test samples or errors in the measurement process. Hence, it is further confirmed that the constructed models are feasible for predicting the sound absorption performance of PPCS.

Figure 4a shows that the absorption coefficient of the PPCS with 30 mm thick polyurethane is larger than that of the SPL with the same thickness in the frequency range of 200–1600 Hz. Furthermore, an almost perfect absorption coefficient appears at about 880 Hz under the resonance effect of PR, and the thickness of PPCS is about 1/13 of the wavelength at this frequency. The average absorption coefficient of the SPM is 0.33, while that of the PPCS is 0.53, that is, the sound absorption of PPCS is approximately 60% better than that of SPL in the same frequency range. Similarly, Figure 4b also shows that the absorption coefficient of the PPCS with 40 mm thick melamine is larger than that of the SPM with the same thickness in 200–1600 Hz: the average absorption coefficient of the PPCS is 0.76, and the average absorption coefficient of the SPL is 0.66. The first maximum absorption coefficient of the PPCS occurs at 570 Hz, and its thickness is about 1/15 of the corresponding wavelength.

Moreover, in the frequency range below the quarter-wavelength resonance, as shown in the insets in Figure 4a,b, for the PPCSs with thicknesses of 30 mm and 40 mm, their sound absorption coefficients are greater than 0.6 in the frequency ranges of 700–2830 Hz and 425–2125 Hz, respectively, both with effective absorption bandwidths exceeding two octaves. This result indicates that the proposed structure can be recognized as a special type of acoustic metamaterial with broadband sound absorption.

## 3. Results and Discussion

### 3.1. Sound Absorption Mechanism of PPCS

**Figure 5:**
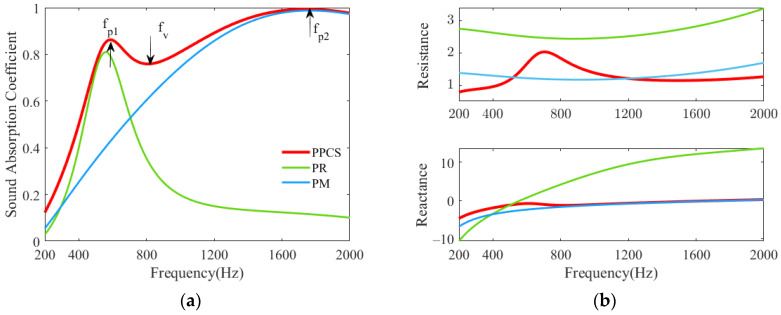
Sound absorption of PPCS and its components PR and PM. (**a**) Sound absorption coefficients; (**b**) normalized surface acoustic impedance.

To explore the underlying sound absorption mechanism of the PPCS, the sound absorption coefficients and acoustic impedances of the composite structure PPCS and the two components PR and PM in the frequency range of 200–2000 Hz are plotted in Figure 5. The porous material is melamine foam, and the structural parameters are the same as those in Table 1.

Figure 5a shows that for the PPCS, there are two absorption peaks at $f_{p1}=585$ Hz and $f_{p2}=1740$ Hz, and one absorption valley at $f_{v}=810$ Hz; for the PR and PM, the absorption peaks are located at 560 Hz and 1770 Hz, respectively. The two resonant frequencies of the PPCS correspond to those of PR and PM, respectively, and the slight deviations between them may be caused by the coupling of the acoustic reactance of both components. Furthermore, Figure 5b reveals that the relatively large acoustic resistance of PPCS is similar to that of the porous material except for at the frequencies in the vicinity of the absorption valley. Importantly, an acoustic reactance close to zero can be achieved particularly at the frequencies below the absorption valley because of the partial perforation introduced into the porous-material substrate. Therefore, it can be interpreted that the sound absorption at the lower frequencies (below the valley frequency) is mainly attributed to the resonance of PR; the porous material is responsible for the sound absorption at the higher frequencies (above the valley frequency); the effective sound absorption at the valley frequency originates from the strong coupling mode between the PR and PM.

**Figure 6:**
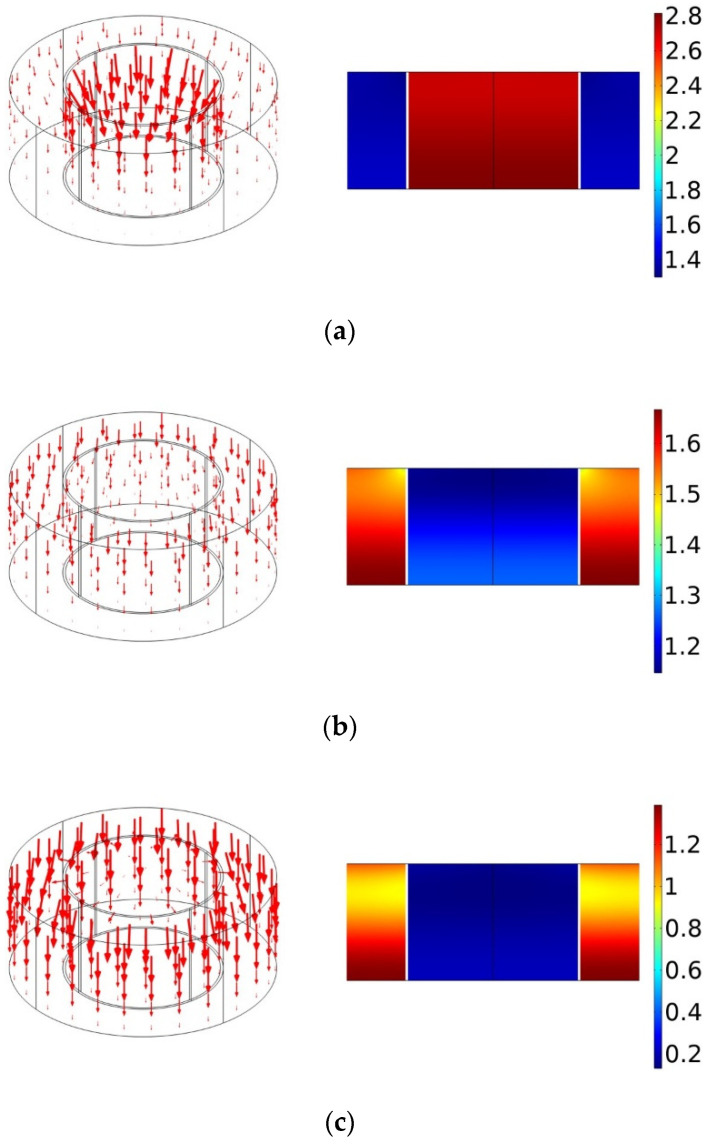
The distribution of particle velocity (left) and normalized sound pressure (right) at frequencies of (**a**) $f_{p1}=585$ Hz, (**b**) $f_{v}=810$ Hz, (**c**) $f_{p2}=1740$ Hz.

In addition, Figure 6a–c shows the distributions of particle velocity and normalized sound pressure in the PPCS at the peak and valley frequencies. At the first peak frequency $f_{p1}=585$ Hz, most of the particle velocity vectors point towards the upper surface of the PR, i.e., the perforated plate; the maximum acoustic pressure is located in the PR, with an amplitude of about 2.8 times of the incident acoustic pressure. This is because the low-frequency acoustic waves with large wavelengths can easily penetrate through the perforated plate and then reach the porous layer in the cavity of the PR. On the other hand, the particle velocity flow in the PM is very weak and the normalized acoustic pressure is almost close to that in the incident field, that is, the PM can hardly absorb the acoustic energy at low frequencies. At the second peak frequency $f_{p2}=1740$ Hz, the particle velocity flow is mainly distributed in the PM, and the maximum normalized sound pressure is observed at the bottom of the PM. This is because the high-frequency acoustic waves with a small wavelength are easily trapped in the porous material. In addition, at this frequency, the acoustic wave is almost completely reflected on the upper surface of the PR, so the normalized pressure in the cavity of the PR is small. Therefore, it can be concluded that the PR and PM play a dominant role in the sound absorption at the first and second peaks, respectively. At the valley frequency $f_{v}=810$ Hz, the particle velocity flow seems to be uniformly distributed in the PR and PM, and the normalized pressure at this frequency is between those at $f_{p1}=585$ Hz and $f_{p2}=1740$ Hz. This also confirms that the sound absorption enhancement of PPCS is not only attributed to the resonance mode of PR at lower frequencies and the capture mode of PM at higher frequencies but also results from the strong coupling mode between the PR and PM, which effectively expands the absorption bandwidth.

### 3.2. Comparison of Sound Absorption between PPCS and Other Structures

**Figure 7:**
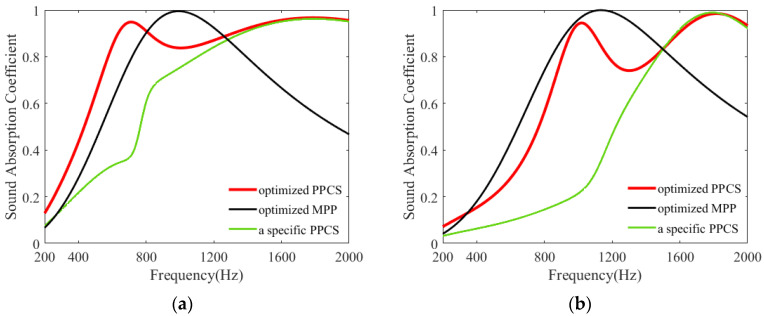
Sound absorption coefficients of optimized PPCS, optimized MPP, and a specific PPCS (the same PPCS structure without porous-material filling in the PR). (**a**) Porous materials of melamine with a thickness of 40 mm; (**b**) porous materials of polyurethane with a thickness of 30 mm.

To illustrate the feasibility of the proposed PPCS for sound absorption at low–mid frequencies, the absorption coefficients of the PPCS and two other structures are given in Figure 7. The other two structures used for comparison are the classical MPP absorber and a special PPCS (the same PPCS absorber without porous-material filling in the PR). In Figure 7a,b, the porous-material substrates in the PPCSs are melamine with a thickness of 40 mm and polyurethane with a thickness of 30 mm, respectively, and their five non-acoustic parameters are the same as those in Table 1. For the above given porous materials, the structural parameters of PR are optimized using the genetic algorithm [43,44,45], such as the hole diameter *d*, perforation rate *p*, and its occupied area ratio A_PR_ in the PPCS. The geometric parameters of the optimized PPCS and optimized MPP are listed in Table 2.

Firstly, MPP and PPCS with the same space dimensions are used for comparison in the frequency range of 200–2000 Hz. As observed in Figure 7a, for the optimized PPCS with a diameter of 5.1 mm and perforation rate of 2.08%, it has a relatively higher average sound absorption coefficient of 0.81; while for optimized MPP with a diameter of 0.3 mm and perforation rate of 4.50%, its average absorption coefficient is about 0.67. Likewise, in Figure 7b, for optimized PPCS with a diameter of 5.5 mm and perforation rate of 4.15%, it has an average sound absorption coefficient is about 0.64; and for optimized MPP with a diameter of 0.3 mm and perforation rate of 4.15%, its average absorption coefficient is about 0.65. Therefore, it can be concluded that PPCS with a large diameter and low perforation rate has the potential to achieve the sound absorption performance of MPP at specific frequencies.

Moreover, as plotted in Figure 7a, in comparison with the PPCS without porous-material filling in the PR, the PPCS with the same configuration but with porous-material filling in the PR has a distinct absorption peak at 705 Hz and its absorption coefficient is significantly higher in the frequency range of 200–1450 Hz. Similarly, in Figure 7b, the sound absorption coefficient of PPCS is also larger than that of the specific PPCS in the frequency range of 200–1485 Hz, and there is also an absorption peak located at 1020 Hz. That is, because of the addition of porous materials in the cavity of the PR, not only does the acoustic resistance of PR increase but also its stiffness becomes smaller, thus, PR has an obvious resonance dissipation at low frequencies.

### 3.3. Influence of Parameters on Sound Absorption of PPCS

**Figure 8:**
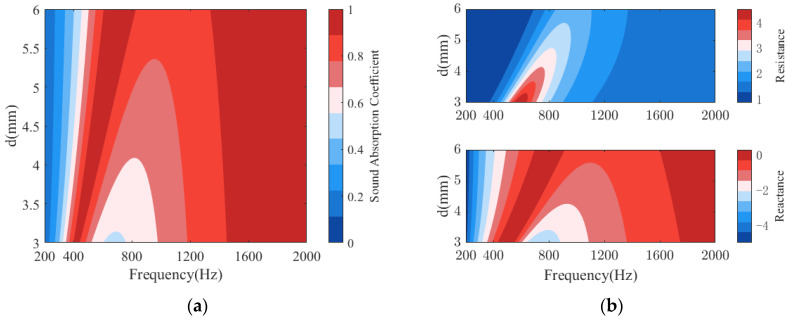
Effects of the hole diameter d on the sound absorption of PPCS: (**a**) sound absorption coefficients; (**b**) normalized surface acoustic impedance.

Figure 8 plots the sound absorption of the PPCSs with different hole diameters *d*. As shown in Figure 8a, for a given perforation spacing, as the hole diameter increases, especially in the low-frequency range controlled by the PR, the effective sound absorption gradually moves to higher frequencies, and the bandwidth of perfect absorption increases, with sound absorption coefficient close to 1; the absorption valley occurs at higher frequencies and the coupling mode between the PR and PM is enhanced so the corresponding absorption coefficient increases; in the high-frequency domain dominated by the PM, the bandwidth of perfect absorption becomes wider and shifts to lower frequencies. This variation can be explained by the acoustic impedance in Figure 8b, where a distinct feature is that the acoustic resistance at the valley frequency changes from an excessive resistance to a suitable resistance close to 1 as the hole diameter increases. In addition, the trend of the acoustic reactance is similar to that of the absorption coefficient, with the increase of the hole diameter, the acoustic reactance value approaches 0, thus there is a higher absorption for PPCS.

**Figure 9:**
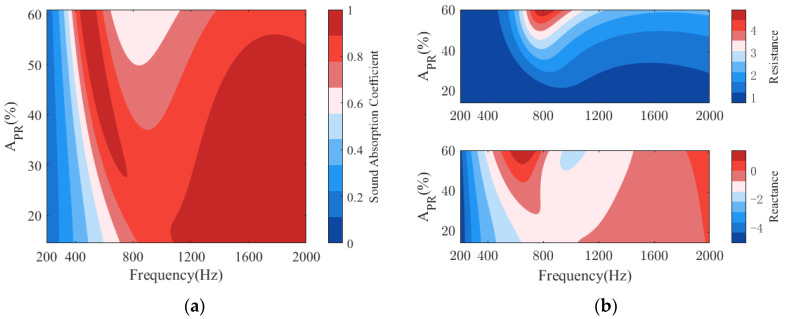
Effects of the area occupancy ratio *A_PR_* of PR on the sound absorption of PPCS: (**a**) sound absorption coefficients; (**b**) normalized surface acoustic impedance.

Figure 9 plots the absorption of PPCS for different area occupancy ratios APR of the PR. As seen in Figure 9a, when the area ratio is varied from 15% to 61%, there is a relatively observable change in sound absorption in the low-frequency domain contributed by the PR, with the increased bandwidth of perfect absorption occurring at lower frequencies. Although the absorption in the high-frequency domain is somewhat reduced, it remains as effective. It is easy to understand that the increased area occupancy ratio of PR will make the PR dominate the sound absorption of PPCS, especially at low frequencies. In addition, the absorption coefficient at high frequencies remains effective despite some reduction. This can also be explained according to the acoustic impedance in Figure 9b. For example, in the frequency range of 400–800 Hz, the acoustic resistance of PPCS varies from 1 to 3, and the acoustic reactance changes from −2.5 to 1 with the increase of the area ratio of PR, so the PPCS has a relatively moderate acoustic impedance, resulting in relatively satisfactory sound absorption in this frequency range. However, as the area ratio increases, the absorption coefficient at the valley frequency declines because as the area ratio of PR increases, the acoustic resistance and reactance at the valley frequency vary from 0.85 to 4.5 and −1.5 to 1 and the acoustic impedance of the PPCS no longer matches that of the air. Therefore, an appropriate area ratio can not only bring perfect absorption with an absorption coefficient close to 1 at low and high frequencies, but also ensure effective absorption with the minimum absorption coefficient at valley frequencies greater than 0.6.

**Figure 10:**
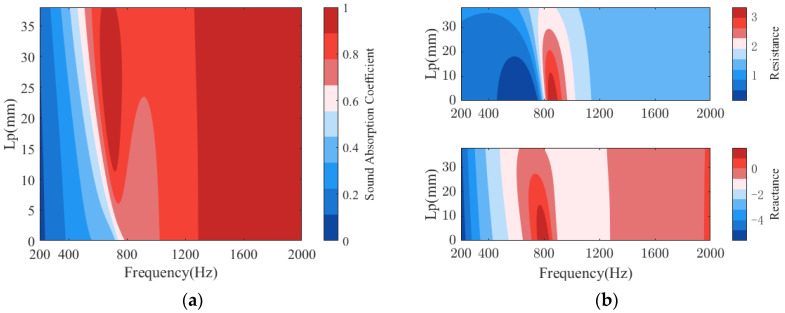
Effects of the porous-material thickness *Lp* in the cavity of PR on the sound absorption of PPCS: (**a**) sound absorption coefficients; (**b**) normalized surface acoustic impedance.

Figure 10 shows the sound absorption of PPCS with different thicknesses *Lp* of the porous-material filling in the cavity of the PR. The thickness *Lp* mainly affects the sound absorption of PPCS in the low-frequency domain, which is dominantly the sound absorption band of PR. With the increase of *Lp*, the sound absorption band of PR shifts to lower frequencies, and the perfect absorption bandwidth increases first and then decreases. This is because the stiffness of PR decreases with the addition of porous material filling, so the resonance occurs at lower frequencies. However, it should be noted that too much porous material may cause excessive sound resistance and thus reduce sound absorption. The sound absorption of PPCS with and without porous material filling in the cavity of the PR compared in Section 3.2 are two special cases, and their acoustic properties are similar to those in this section. Moreover, the addition of porous materials in the PR has little effect on sound absorption in the high-frequency domain. In other words, it is necessary to consider the introduction of porous-material filling in the PR for the enhancement of low-frequency sound absorption of PPCS.

**Figure 11:**
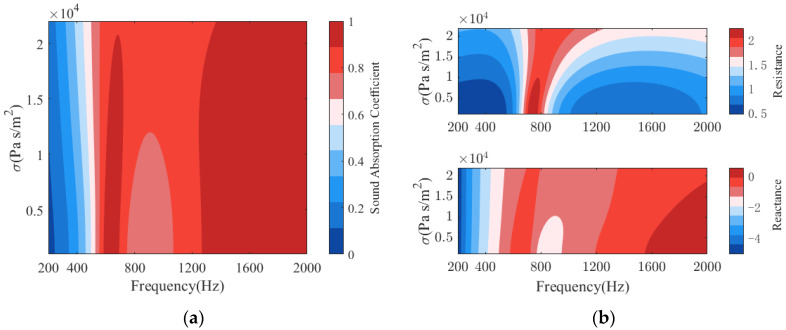
Effects of the flow resistance of PM on the sound absorption of PPCS: (**a**) sound absorption coefficients; (**b**) normalized surface acoustic impedance.

Figure 11 displays the sound absorption of PPCS with different flow resistances of porous materials. From Figure 11b, it can be found that when the flow resistance is 1000–20,000 Pas/m^2^, the perfect absorption slightly shifts to the higher frequency in both low-frequency domain and high-frequency domain, and the sound absorption increases at the valley frequency; even when the flow resistance is higher than 20,000 Pas/m^2^, the sound absorption coefficients are large in the low-frequency domain. Similarly, as observed in Figure 11b, in the frequency range of 700–1200 Hz, the values of sound resistance and acoustic resistance are in the ranges of 1.7 to 2.2 and −0.8 to −1.5, respectively, and they change relatively slowly. In other words, the effect of flow resistance on the sound absorption of PPCS is inconspicuous, and the proposed PPCS can effectively improve the low-frequency sound absorption of porous materials with a wide range of flow resistances.

## 4. Conclusions

In this work, a novel composite structure of porous material mosaicked with perforated resonators is proposed to enhance the sound absorption performance of a single porous layer at low frequencies. Theoretical and numerical models are established to predict the acoustic properties of the proposed structure, and two samples made of polyurethane and melamine, respectively, are tested for experimental validation. The underlying mechanism of the sound absorption of this structure is illustrated through comparisons of the acoustic impedances of the structure and its two components (the PR and PM), as well as the distributions of particle velocities and sound pressure at particular frequencies. In addition, the optimal sound absorption of the proposed structure is compared with that of the classical micro-perforated panel in the same frequency range. Finally, the effects of the structural parameters on the sound absorption of the proposed structure are analyzed. Based on the results obtained in these investigations, the main conclusions are drawn as follows:

(1) The PR filled with porous material in the back cavity is conducive to excite coupled modes and to achieve a high sound absorption in a wide frequency band, and this proposed structure is a broadband acoustic metamaterial. In the frequency range below the quarter-wavelength resonance, the samples with thicknesses of 30 mm and 40 mm have sound absorption coefficients greater than 0.6 in 700–2830 Hz and 425–2125 Hz, respectively. Both demonstrate effective absorption in bandwidths more than two octaves.
(2) The proposed composite structure has acoustic performance equivalent to the classical MPP and can be used as an alternative to the MPP for low–mid frequency sound absorption. In the specific frequency range of 200–2000 Hz, for a space thickness of 40 mm, the optimal average sound absorption coefficients of the structure with hole diameter of 5.1 mm and the MPP with hole diameter of 0.3 mm are 0.81 and 0.67, respectively; for a space thickness of 30 mm, the optimal average sound absorption coefficients of the structure with hole diameter of 5.5 mm and the MPP with hole diameter of 0.3 mm are 0.64 and 0.65, respectively.
(3) The result of parameter analysis shows that the hole diameter, the thickness of porous material in the cavity of PR, and the area occupancy ratio have significant effects on the sound absorption characteristics of the proposed structure, especially at low frequencies, while the flow resistance of the porous material has a mild effect on the sound absorption characteristics.

In conclusion, the proposed method of mosaicking perforated resonators with regular hole diameters can effectively enhance the sound absorption of porous material in the low-frequency range. It will be beneficial for low frequency noise reduction in cars, home appliances and indoor acoustics. In the next work, the sound absorption characteristics of PPCS in the diffuse sound field will be concerned and studied.

## Figures and Tables

**Table 1:** Parameters of PPCS Samples.

Porous-Material Layer of Polyurethane					
$\sigma \;(Pa\cdot s/m^{2})$	ϕ (%)	Ʌ (μm)	Ʌ′ (μm)	$\alpha_{\infty }$	*L* (mm)
2156	0.98	179	318	1.1	40
*d* (mm)	*t* (mm)	*p* (%)	*d*_0_ (mm)	*d_m_* (mm)	*t_p_* (mm)
3.6	2	1.56	100	58	1
**Porous-Material Layer of Melamine**					
$\sigma \;(Pa\cdot s/m^{2})$	ϕ (%)	Ʌ (μm)	Ʌ′ (μm)	$\alpha_{\infty }$	*L* (mm)
15,734	0.99	92	197	1.04	40
*d* (mm)	*t* (mm)	*p* (%)	*d*_0_ (mm)	*d_m_* (mm)	*t_p_* (mm)
4	2	0.96	100	58	1

**Table 2:** Optimized parameters of PPCS and MPP.

Thickness: 40 mm	*d* (mm)	*p* (%)	*A_PR_* (%)	*t* (mm)
PPCS	5.1	2.08	41.5	2
MPP	0.3	4.50	-	2
**Thickness: 30 mm**	***d* (mm)**	***p* (%)**	***A_PR_* (%)**	***t* (mm)**
PPCS	5.5	4.54	55.4	2
MPP	0.3	4.15	-	2

## Data Availability

Not applicable.
